# Evaluating the impact of COVID-19 outbreak on hepatitis B and forecasting the epidemiological trend in mainland China: a causal analysis

**DOI:** 10.1186/s12889-023-17587-3

**Published:** 2024-01-02

**Authors:** Chao-Qun He, Bai-Hong Sun, Wang-Tao Yu, Shu-Yi An, Bao-Jun Qiao, Wei Wu

**Affiliations:** 1 Department of Epidemiology, School of Public Health, China Medical University, Shenyang, Liaoning China; 2 Liaoning Provincial Centers for Disease Control and Prevention, Shenyang, Liaoning China

**Keywords:** COVID-19, Hepatitis B, BSTS, Causal impact, Forecasting

## Abstract

**Background:**

It is uncertain how COVID-19 outbreak influences the hepatitis B epidemics. This study aims to evaluate the effects on hepatitis B owing to the COVID-19 outbreak and forecast the hepatitis B epidemiological trend in mainland China to speed up the course of the “End viral hepatitis Strategy”.

**Methods:**

We estimated the causal impacts and created a forecast through adopting monthly notifications of hepatitis B each year from 2005 to 2020 in mainland China using the Bayesian structural time series (BSTS) method.

**Results:**

The hepatitis B epidemics fluctuates irregularly during the period 2005–2007(APC = 8.7, *P* = 0.246) and 2015–2020(APC = 1.7, *P* = 0.290), and there is a downturn (APC=-3.2, 95% CI -5.2 to -1.2, *P* = 0.006) from 2007 to 2015 in mainland China. The COVID-19 outbreak was found to have a monthly average reduction on the hepatitis B epidemics of 26% (95% CI 18–35%) within the first three months in 2020,17% (95% CI 7.7–26%) within the first six months in 2020, and 10% (95% CI19–22%) all year as a result of the COVID-19 outbreak, (probability of causal effect = 96.591%, *P* = 0.034) and the forecasts showed an upward trend from 2021 to 2025 (annual percentage change = 4.18, 95% CI 4.0 to 4.3, *P* < 0.001).

**Conclusion:**

The COVID-19 has a positive effect on the decline of hepatitis B cases. And the potential of BSTS model to forecast the epidemiological trend of the hepatitis B can be applied in automatic public health policymaking in mainland China.

**Supplementary Information:**

The online version contains supplementary material available at 10.1186/s12889-023-17587-3.

## Background

Viral hepatitis B (hereinafter referred to as hepatitis B) is an infectious diseases caused by hepatitis B virus (HBV) characterized by liver damage [1]. Patients and HBV carriers are the main sources of hepatitis B infection [2], and is mainly transmitted through infected blood and body fluids, sexual contact, and mother-to-child transmission [3, 4]. Hepatitis B is classified into acute, chronic, and undifferentiated types, with chronic cases potentially leading to cirrhosis or hepatocellular carcinoma [5], and ultimately die of liver failure and other complications in the end [6]. The disease is a significant public health threat, with approximately 820,000 deaths worldwide reported in 2019 and almost 296,000,000 people living with HBV globally claimed by the World Health Organization (WHO) [7]. China has the highest burden of hepatitis B disease in the world [8, 9], with the number of reported cases remaining among the highest of notifiable infectious diseases in mainland China over the past few decades. China has implemented various prevention and control measures to eliminate the disease, including vaccination, interrupting mother-to-child transmission, and ensuring blood safety [9, 10]. However, an estimated 84 million people worldwide remain infected, which is more than the entire population of any European country. The outbreak of COVID-19 in early 2020 has led to significant health, economic, and social impacts, causing a decrease in responses to non-emergency services due to an overburdened healthcare system, as well as concerns about infection during medical treatment [11–13] The pandemic has also affected the implementation of hepatitis B prevention and treatment plans. It is crucial to estimate how the COVID-19 outbreak affects the reduction in hepatitis B cases and forecast the epidemiological trends in mainland China to guide the formation of epidemic prevention policies related to hepatitis B and achieve the “End Viral Hepatitis Strategy” by 2030 [14].

Accurate forecasting relies on accurate data statistics. In previous studies, conventional statistical models were used to estimate the diseases epidemic patterns, such as the Grey Model and Autoregressive Integrated Moving Average (ARIMA) model. Compared with other mathematical model methods, the classical grey model GM (1,1) is essentially a homogenous exponent model, due to the grey action quantity is a constant number. It is quite susceptible to external influencing factors, the model accuracy maybe low [15, 16]. The ARIMA model, as a linear model capturing the linear relationship isn’t suitable for hepatitis B incidence trend which tends to be nonlinear characteristics. And mainly used to fit time series with stationary properties (or can be converted to stationary series), so it is limited in certain applications [17, 18]. In this study, we use Bayesian structural time series (BSTS) model to explore how the COVID-19 outbreak effect the hepatitis B epidemic in mainland China. The BSTS method was developed by combing Kalman filters, Spike and slab regression with Bayesian model averaging into an integrated system [19]. The Kalman filters estimate the trend and seasonality of the target series, the Spike and slab regression perform variable selection [20], and the Bayesian model averaging produces a final forecast [21]. The trend, seasonality, and regression components can be studied separately through the stochastic state-space model, and the method can quantify the effects of the COVID-19 outbreak on the hepatitis B epidemic and forecast the epidemiology trends by generating counterfactual predictions in a synthetic control series describing what would have happened without COVID-19. The BSTS model can handle complex covariate structures and incorporate empirical prior information about parameters to infer patterns of change in potential effects [22]. The predictions produced by BSTS models rarely rely on certain hypothetical norms [18] and have been widely used in many fields, including medicine, economics, marketing management, and meteorology [23–26].

This study set out to estimate the influence of the COVID-19 on the hepatitis B epidemic and to forecast the epidemiological trends in mainland China. The BSTS model is used in this study due to its ability to handle complex covariate structures and produce accurate predictions. The insights generated from this study can guide the formulation of strategies and accelerate progress towards achieving the “End Viral Hepatitis Strategy” by 2030.

## Methods

### Data sources

In this study, we obtained monthly notifications of hepatitis B each year from 2005 to 2020 in mainland China from the National Health of the People’s Republic of China, and extracted the population data from the National Buraeu of statistics in this study.

### Statistical process

We investigated the epidemic pattern of hepatitis B using annual percentage change (APC) through the Joinpoint Regression Program (Version 4.9.1.0). To establish the BSTS model, we used the recently developed bsts package and the CausalImpact package in R is used to evaluate the causal effect in R (Version 4.2.2). BSTS model employed Markov Chain Monte Carlo (MCMC) methods, such as Gibbs sampling, to perform the difficult analytical computation process of the Bayesian posterior distribution [27]. During the course of the BSTS model development, a BSTS model adding a local linear trend and a seasonal to the state specification was the best suitable for our data. We obtained the forecasts with 95% CI by averaging across the 500 MCMC draws using the BSTS model. It has been shown that infectious disease prediction is associated with seasonal patterns [28, 29]. And reported cases are connected with the total population and remain steady even under interventions. Therefore, the seasonal effect, population data, and time variable should be considered as covariates in the causal impact analysis based on the BSTS model. Since January 2020, rigorous prevention and control measures (i.e., maintain social distance, nucleic acid test, stay-at-home order, two-week observation period and etc.) have been taken in mainland China in response to the COVID-19 pandemic [12, 30]. In the meantime, these measures have been adjusted (in other words, these measures have been lifted or relaxed) in different stages depending on the evolution of the epidemic. So, we regarded 2020 as the intervention while performing an intervention analysis using the BSTS model. Then, the BSTS method predicted the expected cases under a counterfactual framework (i.e., there were no COVID-19), and then compared the resulting forecasts with the actual data. In terms of the COVID-19 pandemic influence on the disease patterns [31, 32], we divided the time series into nine in-sample training horizons(2005.01-2019.12, 2005.01-2018.12, 2005.01-2017.12, 2005.01-2016.12, 2005.01-2015.12, 2005.01-2014.12, 2005.01-2013.12, 2005.01-2012.12, 2005.01-2011.12) and out-of-sample validation horizons(the remaining data) based on the periodic behaviors of the hepatitis B cases. Finally, we re-developed the BSTS model based on the 16 years of data to project into 2025.

The statistical formula for a Bayesian structure time series model is shown below, where y represents the observed data, z represents the state variable, β represents the regression component, and ε represents the random error term.

1 $$\begin{array}{c}{y}_{t,j}={z}_{{t}^{\alpha }t}^{T}+{\beta }^{T}{x}_{t,j}+{\epsilon}_{t,j}\end{array}$$

We measured the accuracy of the model in forecasting the hepatitis B epidemiological trends using the mean absolute error (MAE), root-mean-square error (RMSE), and mean absolute percentage error (MAPE) as follows: ${X_i}$, $\widehat {{{X_i}}}$ and *N*, is the observed values, the forecasting values and the number of forecasts, respectively.

2 $$MAE=\frac{1}{N}\sum\nolimits_{{i=1}}^{N} {\left| {{X_i} - \widehat {{{X_i}}}} \right|} $$

3 $$RMSE=\sqrt {\frac{1}{N}\sum\nolimits_{{i=1}}^{N} {{{\left( {{X_i} - \widehat {{{X_i}}}} \right)}^2}} } $$

4 $$MAPE=\frac{1}{N}\sum\nolimits_{{i=1}}^{N} {\frac{{\left| {{X_i} - \widehat {{{X_i}}}} \right|}}{{{X_i}}}} \times 100$$

## Results

### Descriptive statistics

**Fig. 1:**
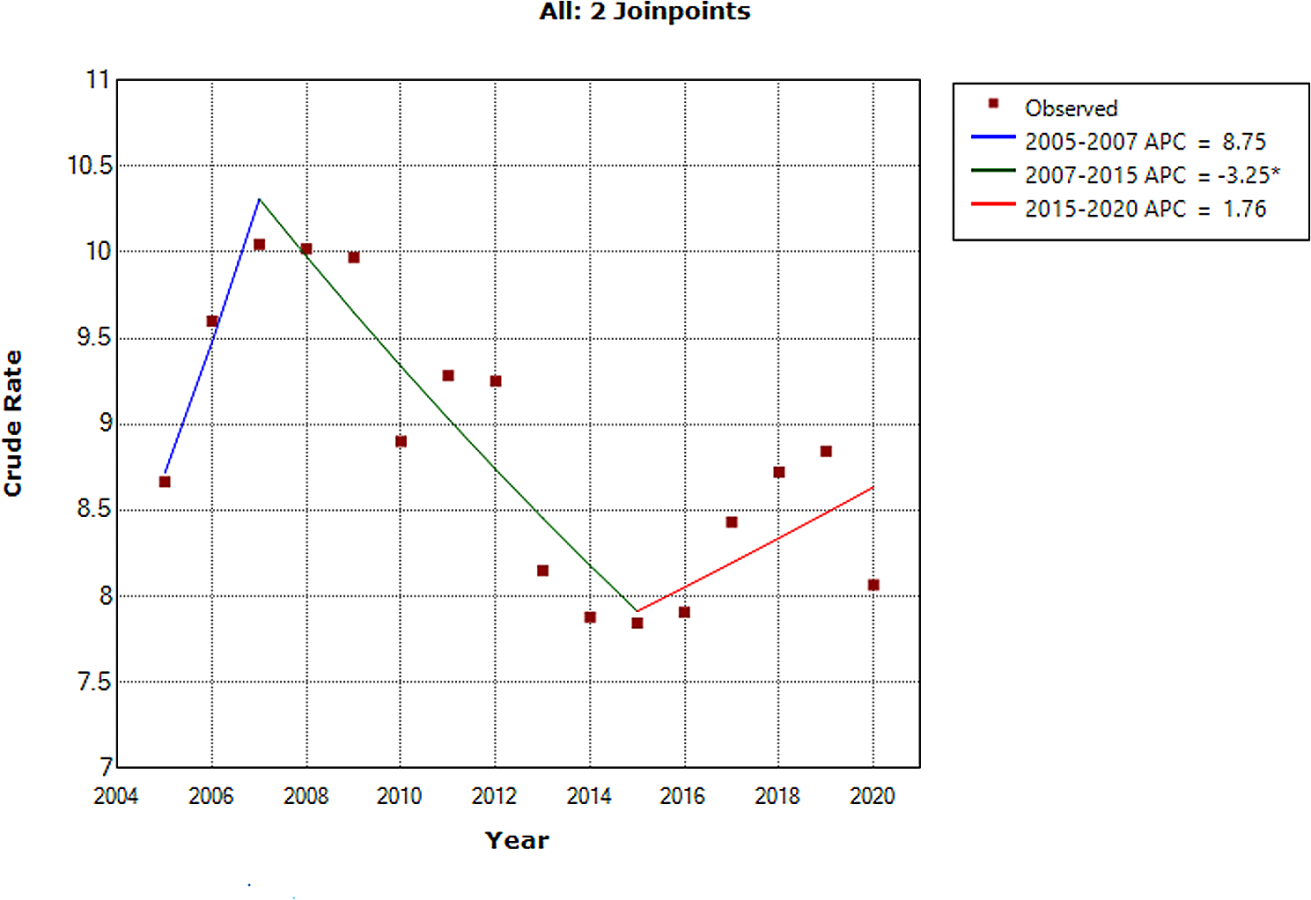
Joinpoint regression displaying the hepatitis B epidemics during the period 2005–2020. *Shows significant difference

During the period of 2005–2020, a total of 23,911,265 cases of hepatitis B were reported in mainland China, refers to an average notification of 124,537 and an annualized average incidence of 8.79 per 10,000 persons. The largest number of cases notified in 2008, 133,0654 cases (10.04 cases per 10,000 people), and the lowest number of cases notified in 2014, 1038,924 cases (7.88 cases per 10,000 people) (Table S1 and Table S2). The hepatitis B epidemics fluctuates irregularly during the period 2005–2007(APC = 8.7, *P* = 0.246) and 2015–2020(APC = 1.7, *P* = 0.290), and there is a downturn (APC=-3.2, 95% CI -5.2 to -1.2, *P* = 0.006) from 2007 to 2015 in mainland China (Fig. 1).

**Fig. 2:**
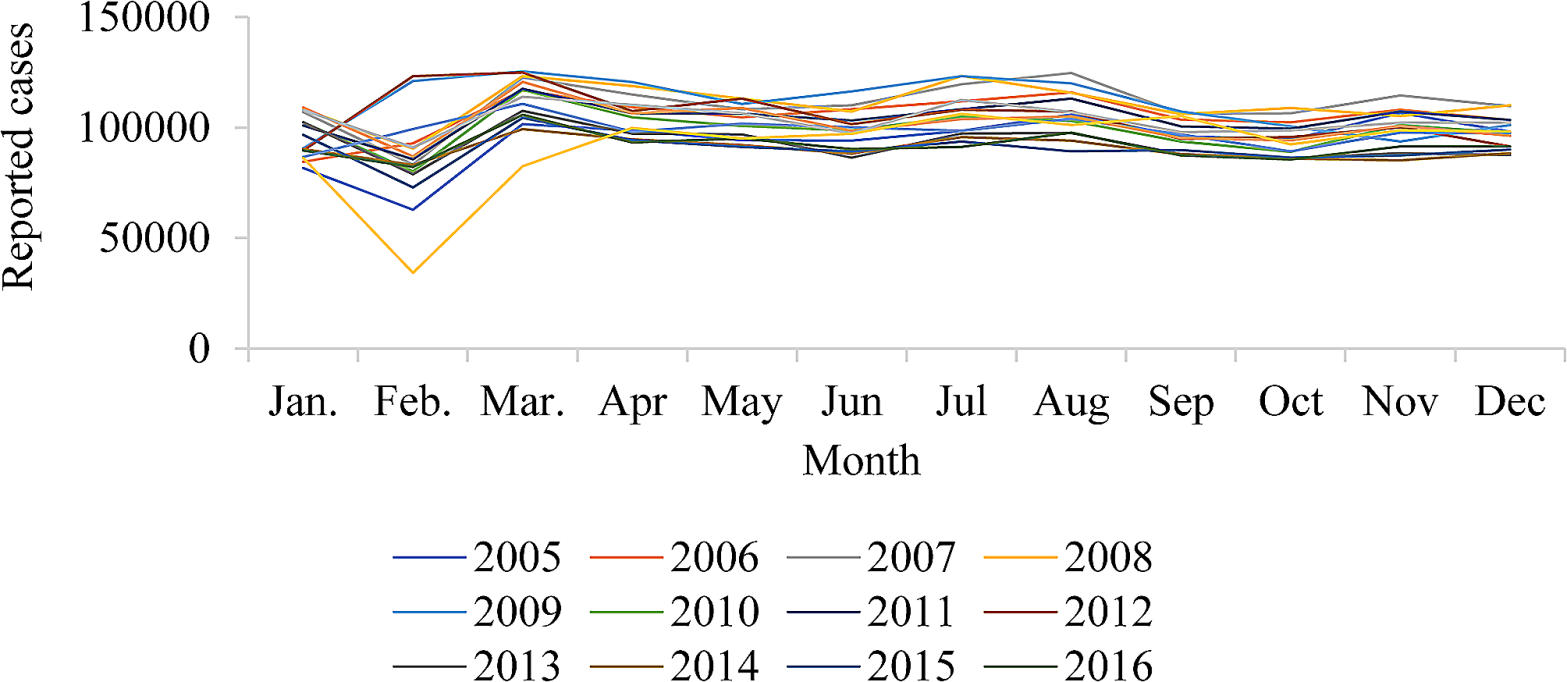
Change in monthly cases of hepatitis B each year from 2005 to 2020 in mainland China

Figure 2 demonstrates that hepatitis B has significant seasonal activity, with the lowest value occurring in February and a rapid rise in the following months from 2005 to 2020.

### Causal analysis

**Fig. 3:**
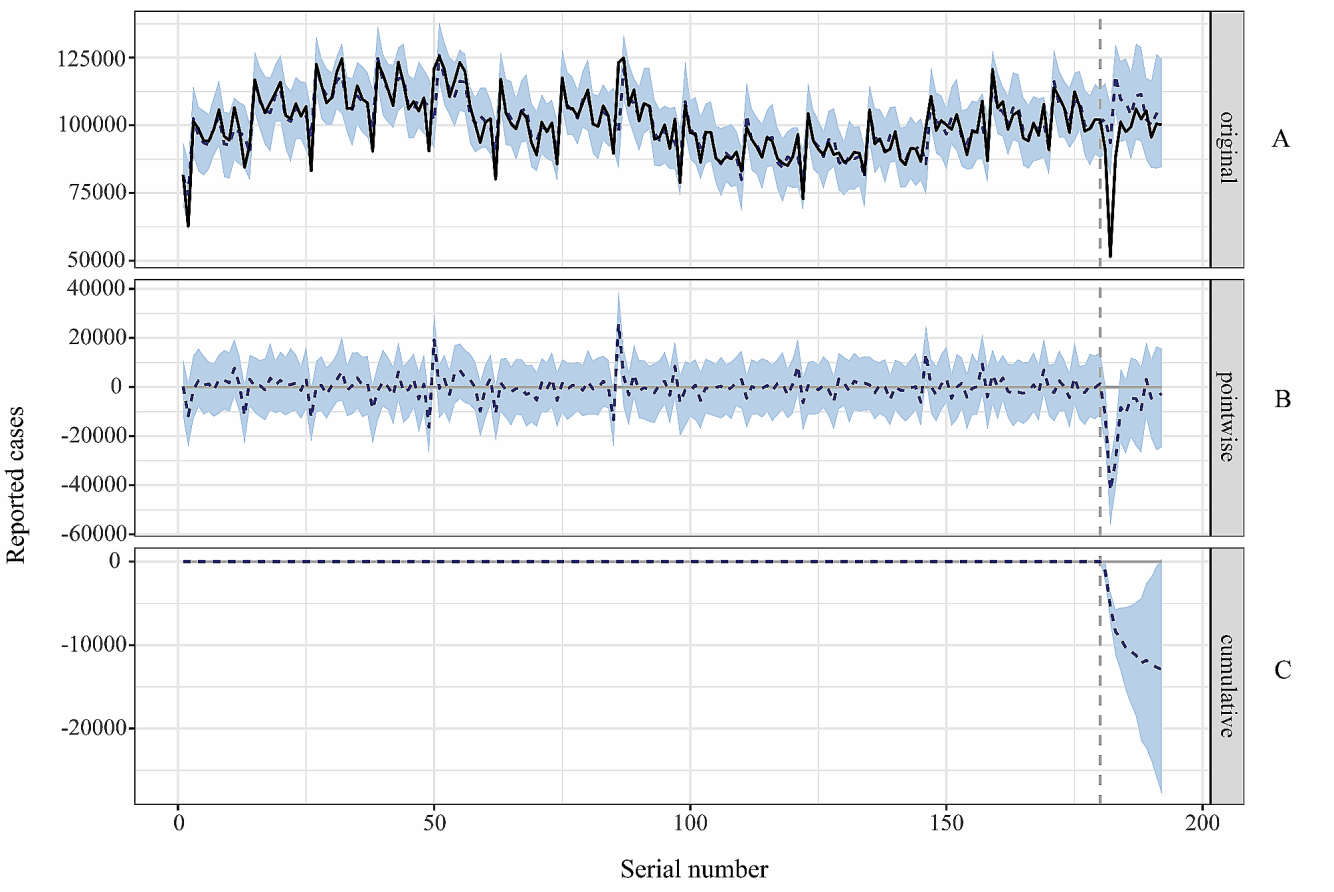
Time series plot of causal effects of the COVID-19 on hepatitis B notifications in 2020. The panel **yA**, **B** and **C** shows the hepatitis B case notifications and counterfactual forecasted results for the post-outbreak period, pointwise causal effect and cumulative effect respectivel

The generated expected figures are listed in Table S3. The effect of COVID-19 on hepatitis B changes with an average reduction of 26% (95% CI 18–35%) within the first three months in 2020,17% (95% CI 7.7–26%) within the first six months in 2020, and 10% (95% CI19–22%) all year. More importantly, the validity of the generated predictions is studied in terms of posterior probability and causal effect probability. In Table S3, it highlights that the posterior probability of causing these effects can be rejected as a random event, while the probability of causal effects can be accepted, pointing to the significant contribution of the COVID-19 outbreak to the reduction in notification of cases of hepatitis B (Fig. 3).

### Forecasting

Table 1 presents the forecasting accuracy level under the BSTS method. To explore whether China could reach the 2030 targets of the End Viral Hepatitis Threat on course, the BSTS model was re-developed to project into 2025 using 16 years of data.

**Table 1:** Measures of BSTS performance

Testing Horizons	MAE	MAPE	RMSE
12-step ahead prediction	4395.847	4.176	5622.687
24-step ahead prediction	4821.816	4.578	5938.697
36-step ahead prediction	4674.154	4.495	5826.284
48-step ahead prediction	6140.172	5.901	7335.529
60-step ahead prediction	7880.740	7.659	9416.390
72-step ahead prediction	14235.330	13.955	17151.620
84-step ahead prediction	32750.720	32.465	39170.760
96-step ahead prediction	33881.080	33.661	42252.210
108-step ahead prediction	27384.980	28.328	28872.140

**Fig. 4:**
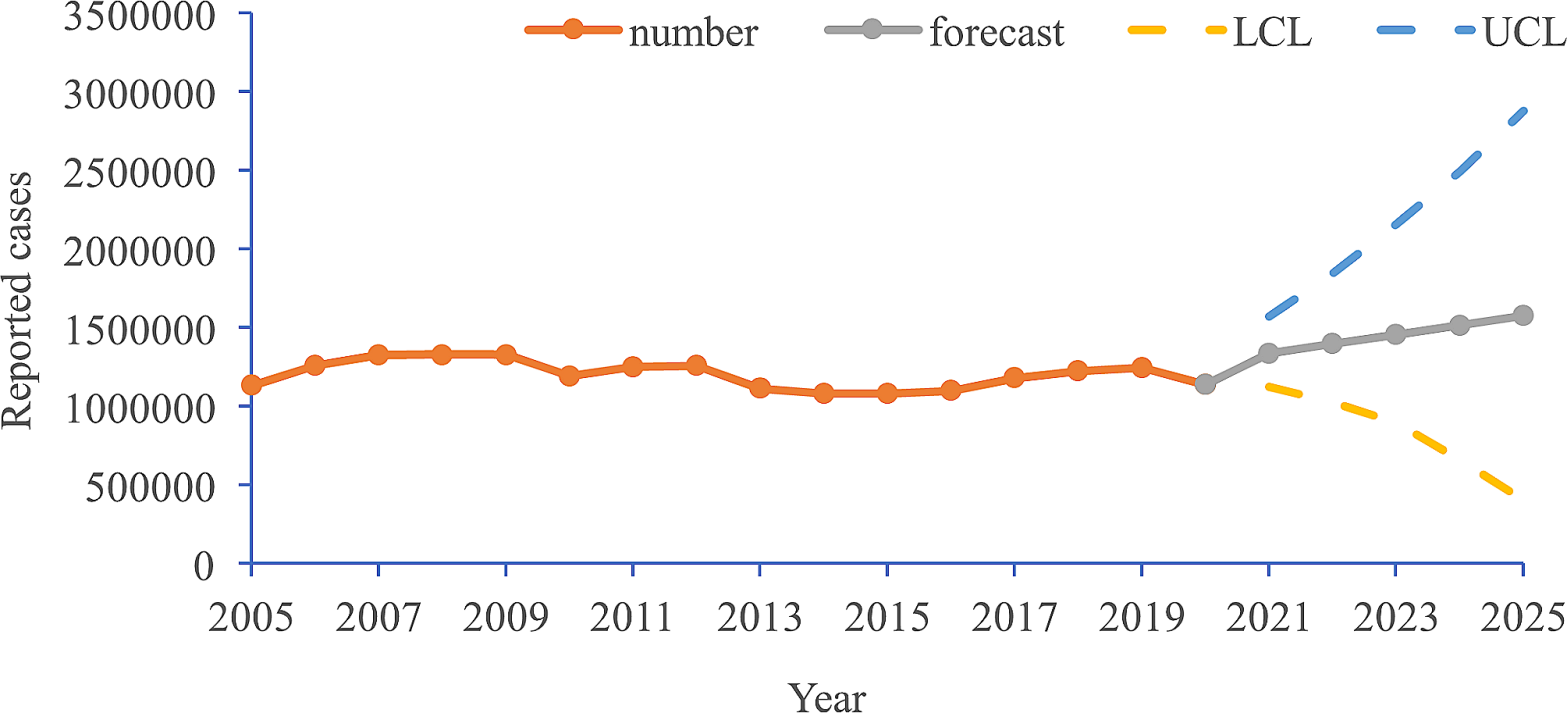
Projections of the hepatitis B reported cases into 2025 based on the BSTS

**Fig. 5:**
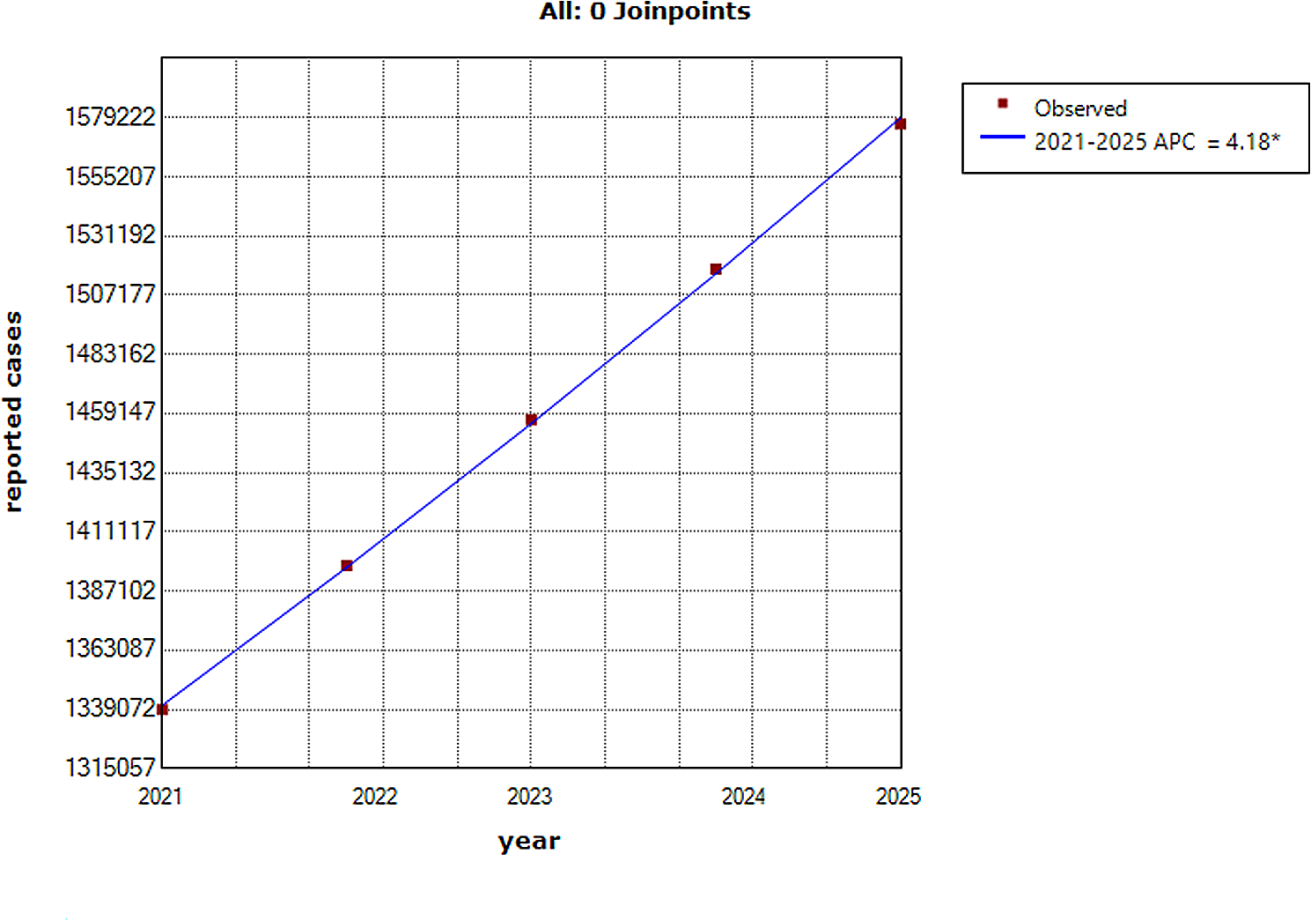
Joinpoint regression displaying the hepatitis B epidemics during the period 2021–2025. *Shows significant difference

The forecasts are reported in Figs. 4 and 5, showing an upward trend (APC = 4.18, 95% CI 4.0 to 4.3, *P* < 0.001) from 2021 to 2025.

## Discussion

The COVID-19 has broken in Wuhan since the beginning of 2020, it gradually evolved into a global public health emergency with its high infectivity and rapid spread speed, causing a series of health, economic and social impacts [12, 33]. During this period, governments worldwide have implemented various prevention interventions [34–36], although which have helped neutralize some of the short-term impacts of the pandemic, the COVID-19 may still influence disease patterns to a certain extent. It is therefore urgent to explore the specific impact of such interventions on reported hepatitis B cases. This study uses intervention analysis under the BSTS model to investigate the impact of the epidemiology on the prevalence of hepatitis B in mainland China, estimating the change of reported case notifications under the influence of the COVID-19 outbreak by comparing the observed time series (the actual number of monthly hepatitis B cases after the COVID-19 outbreak) with the BSTS counterfactual time series (if there were no COVID-19, the number of predictable monthly cases) in the same time frame. And it also forecasts whether the prevalence of viral hepatitis in China is expected to achieve WHO’s strategy [37].

The BSTS model is very feasible to work with complex covariates. It can not only extrapolate temporal patterns of some potential effects, but also incorporate empirical prior information on parameters to measure the effect of interventions [18, 38]. More importantly, the BSTS model is useful for obtaining unbiased impact measures and allows for evolving cumulative effects, enabling effective prioritizing, developing, and implementing intervention strategies to respond to adverse health outcomes in public health [39]. According to our research, the posterior probabilities indicated that it is significantly evident for a real causal impact rather than causing these impacts as a random event. Our analyses indicate a monthly average reduction in hepatitis B case notifications of 26% (95% CI 18–35%) within the first three months in 2020,17% (95% CI 7.7–26%) within the first six months in 2020, and 10% (95% CI19–22%) all year as a result of the COVID-19 outbreak. Although the contagiousness of the virus may last longer, the possibility of household exposure to the HBV may increase [40], and the treatment outcomes may worsen [41], which all can offset the effect of strict anti-virus measures. So, the relative effects of COVID-19 on the reported cases in one year is still justified. However, the exact relationship needs to be confirmed by further exploratory studies.

In addition, several studies suggest that the COVID-19 outbreak may have impacted the pattern of diseases, particularly infectious diseases [42]. This may explain the decrease in hepatitis B case notifications during the pandemic. Firstly, in order to alleviate the overcrowding of hospitals and reduce the risk of COVID-19 infection during medical treatment, we do not encourage people with chronic diseases or mild symptoms to go to medical institutions to seek medical assistance. Secondly, the requirement for negative nucleic acid test results and the discomfort of nasopharyngeal swabs may have dissuaded hepatitis B-infected patients from going to hospitals for diagnosis and treatment. Hospitals were also unable to provide non-emergency services timely due to the burden of the healthcare system and reallocation of limited medical staff and diagnostic platforms to the COVID-19 response. Additionally, disruptions to hepatitis B-related health services and facilities due to the pandemic have led to a reduction in diagnosis and treatment services. Intravenous drug abuse is a significant route for blood-borne transmission of infectious diseases, including hepatitis B. Approximately 9.0% (95% UI 5.1–13.2) of people who inject drugs are HBsAg positive, indicating that about 1.4 million (0.7–2.3 million) people are infected with HBV [43]. Due to COVID-19 prevention and control measures, most countries and regions have implemented varying degrees of entry and exit controls, resulting in a decline in cross-regional mobility and drug exchange. Furthermore, reduced gatherings and the risk of drug use have contributed to the decrease in hepatitis B reported cases. These factors have led to reduced access to medical services related to viral hepatitis. Thirdly, strict traffic control measures and different degrees of control measures for production activities have made the procurement and transportation of drugs and laboratory consumables inconvenient and likely to be interrupted, limiting access to medical services. Fourthly, the COVID-19 pandemic has disrupted livelihoods, strained the economic system, and increased employment pressures, leading some patients to delay seeking further diagnosis and treatment for symptoms. Finally, during the COVID-19 pandemic, the recording and reporting of confirmed cases may be delayed. In terms of predictive power, models with MAPE values falling within (5%, 10%) are considered highly accurate; Models with MAPE values within (10%, 20%) are considered good [44]; So, the BSTS model is suitable to forecast the epidemiological trend of hepatitis B [45].

The goal is to eliminate the public health threat of hepatitis B by 2030, however, forecasts indicate an upward trend in the incidence of hepatitis B from 2021 to 2025, which poses significant challenges for hepatitis B prevention and control in mainland China. This trend may be related to several factors: (a) Medical institutions have strengthened the management of infectious disease reporting, and the underreporting rate has decreased significantly; (b) Some people do not produce protective antibodies or the level of antibodies decreases, thereby increasing the risk of infection and the incidence rate; (c) With the continuous development of the social and economic level, population mobility increases, social activities are frequent, and high-risk individuals are at an increased risk of herd transmission; (d) It may be caused by repeated reporting of chronic hepatitis B cases. For patients with chronic hepatitis B who have been treated repeatedly, medical institutions have repeatedly reported cases in order to prevent underreporting and late reporting. To further improve the prevention and control measures of hepatitis B, the following points should be considered: firstly, we should strengthen the publicity and education work about hepatitis B, focusing on farmers, young adults and men with high incidence. This includes actively advocating that the public fully and systematically understand the relevant knowledge of hepatitis B, improving awareness of hepatitis B protection, and insisting on personal protection to reduce the incidence of high-risk groups. Secondly, it is important to strengthen public recognition of the necessity of hepatitis B vaccination, so that people can actively receive the hepatitis B vaccine. Finally, more policy support should be given to areas with a high incidence of hepatitis B, with precise policies applied for different regions due to the difference in incidence. This includes implementing neonatal vaccination, improving the timely first dose vaccination rate, and optimizing and adjusting vaccine immunization program according to the epidemic characteristics of hepatitis B to increase the vaccination coverage of special populations [46]. Lastly, it is crucial to ensure the authenticity and effectiveness of the disease incidence data by rigorously performing statistical analysis of the reported data of statutory infectious diseases in China. This will help prevent errors in the statistical process and present the most accurate and real data for post-production. The exploration and analysis of the incidence of hepatitis B continue to pave the way for the subsequent construction of a reasonable and accurate quantitative system.

### Strengths and limitations

This study aimed to forecast the counterfactual scenario, i.e., what would happen without the COVID-19. While accurate case data are crucial for statistical inference, the limitations of passive surveillance systems make underreporting inevitable. Furthermore, as this research is based on national data, the model needs refinement with local data to enhance its prediction efficiency when applied to other countries or regions.

## Conclusion

COVID-19 have a positive effect on the decline of hepatitis B case notifications. The potential of the BSTS model to forecast the epidemiological trend of hepatitis B is of valuable reference. This model could be applied to automatic public health policymaking, referring to effective and timely public plans in mainland China. By doing so, limited medical resources could be allocated and current interventions prioritized, thereby accelerating progress towards the “End Viral Hepatitis Strategy.”

### Electronic supplementary material

Below is the link to the electronic supplementary material.

Supplementary Material 1

Supplementary Material 2

Supplementary Material 3

Supplementary Material 4

Supplementary Material 5

## Data Availability

We obtained monthly notifications of hepatitis B each year from 2005 to 2020 in mainland China from the National Health of the People’s Republic of China, and extracted the population data from the National Buraeu of statistics in this study. All the data supporting the findings of the work are contained within the manuscript (see Table S2 and Table S4).

## Abbreviations

APC: Annual percentage change
ARIMA: Autoregressive integrated moving average
BSTS: Bayesian structural time series
CI: Confidence interval
GM: Grey model
HBsAg: Hepatitis B surface antigen
HBV: Hepatitis B virus
MAE: Mean absolute error
MAPE: Mean absolute percentage error
MCMC: Markov chain monte carlo
RMSE: Root-mean-square error
