# Supplementary material for: Evaluating the impact of COVID-19 outbreak on hepatitis B and forecasting the epidemiological trend in mainland China: a causal analysis
Source: BMC Public Health. 2024 Jan 2;24:47. doi: 10.1186/s12889-023-17587-3 (PMC10763123; doi:10.1186/s12889-023-17587-3)
Supplement: Supplementary file 1 — Supplementary Material 1 [file 12889_2023_17587_MOESM1_ESM.docx]

**Table S1** The incidence rate of hepatitis B in mainland China from 2005 to 2020.

| Year | Population  (10000) | Reported cases  (n) | Incidence  (1/10000 population) |
| --- | --- | --- | --- |
| 2005 | 130,756 | 1132,805 | 8.66 |
| 2006 | 131,448 | 1261,735 | 9.60 |
| 2007 | 132,129 | 1327,225 | 10.04 |
| 2008 | 132,803 | 1330,654 | 10.02 |
| 2009 | 133,450 | 1330,352 | 9.97 |
| 2010 | 134,091 | 1193,266 | 8.90 |
| 2011 | 134,916 | 1252,236 | 9.28 |
| 2012 | 135,922 | 1257,320 | 9.25 |
| 2013 | 136,726 | 1114,319 | 8.15 |
| 2014 | 137,646 | 1084,543 | 7.88 |
| 2015 | 138,326 | 1085,113 | 7.84 |
| 2016 | 139,232 | 1100,691 | 7.91 |
| 2017 | 140,011 | 1180,545 | 8.43 |
| 2018 | 140,541 | 1225,877 | 8.72 |
| 2019 | 141,008 | 1247,092 | 8.84 |
| 2020 | 141,212 | 1139,133 | 8.07 |
