# Supplementary material for: Evaluating the impact of COVID-19 outbreak on hepatitis B and forecasting the epidemiological trend in mainland China: a causal analysis
Source: BMC Public Health. 2024 Jan 2;24:47. doi: 10.1186/s12889-023-17587-3 (PMC10763123; doi:10.1186/s12889-023-17587-3)
Supplement: Supplementary file 2 — Supplementary Material 2 [file 12889_2023_17587_MOESM2_ESM.docx]

**Table S2** Time series of hepatitis B and COVID-19 notifications, 2005-2020, mainland China.

| Time | hepatitis B | COVID-19 | Time | hepatitis B | COVID-19 | Time | hepatitis B | COVID-19 |
| --- | --- | --- | --- | --- | --- | --- | --- | --- |
| 01-2005 | 81693 | 0 | 05-2010 | 100653 | 0 | 09-2015 | 89806 | 0 |
| 02-2005 | 62794 | 0 | 06-2010 | 98691 | 0 | 10-2015 | 86393 | 0 |
| 03-2005 | 1101475 | 0 | 07-2010 | 105007 | 0 | 11-2015 | 87284 | 0 |
| 04-2005 | 98758 | 0 | 08-2010 | 103030 | 0 | 12-2015 | 90103 | 0 |
| 05-2005 | 94318 | 0 | 09-2010 | 93643 | 0 | 01-2016 | 89699 | 0 |
| 06-2005 | 94136 | 0 | 10-2010 | 89066 | 0 | 02-2016 | 82204 | 0 |
| 07-2005 | 98588 | 0 | 11-2010 | 101234 | 0 | 03-2016 | 105745 | 0 |
| 08-2005 | 105752 | 0 | 12-2010 | 97590 | 0 | 04-2016 | 93190 | 0 |
| 09-2005 | 96004 | 0 | 01-2011 | 100932 | 0 | 05-2016 | 95079 | 0 |
| 10-2005 | 94489 | 0 | 02-2011 | 85649 | 0 | 06-2016 | 90166 | 0 |
| 11-2005 | 106598 | 0 | 03-2011 | 117553 | 0 | 07-2016 | 91219 | 0 |
| 12-2005 | 98200 | 0 | 04-2011 | 106464 | 0 | 08-2016 | 97670 | 0 |
| 01-2006 | 84489 | 0 | 05-2011 | 106263 | 0 | 09-2016 | 87390 | 0 |
| 02-2006 | 92876 | 0 | 06-2011 | 103219 | 0 | 10-2016 | 85480 | 0 |
| 03-2006 | 116831 | 0 | 07-2011 | 108259 | 0 | 11-2016 | 91478 | 0 |
| 04-2006 | 109330 | 0 | 08-2011 | 113093 | 0 | 12-2016 | 91371 | 0 |
| 05-2006 | 104575 | 0 | 09-2011 | 100517 | 0 | 01-2017 | 86657 | 0 |
| 06-2006 | 108312 | 0 | 10-2011 | 99591 | 0 | 02-2017 | 99417 | 0 |
| 07-2006 | 111905 | 0 | 11-2011 | 107208 | 0 | 03-2017 | 110717 | 0 |
| 08-2006 | 115960 | 0 | 12-2011 | 103488 | 0 | 04-2017 | 98123 | 0 |
| 09-2006 | 103665 | 0 | 01-2012 | 89614 | 0 | 05-2017 | 101783 | 0 |
| 10-2006 | 102399 | 0 | 02-2012 | 123239 | 0 | 06-2017 | 100155 | 0 |
| 11-2006 | 108022 | 0 | 03-2012 | 124899 | 0 | 07-2017 | 98501 | 0 |
| 12-2006 | 103371 | 0 | 04-2012 | 107415 | 0 | 08-2017 | 103977 | 0 |
| 01-2007 | 106956 | 0 | 05-2012 | 113147 | 0 | 09-2017 | 96856 | 0 |
| 02-2007 | 83225 | 0 | 06-2012 | 101592 | 0 | 10-2017 | 89105 | 0 |
| 03-2007 | 122566 | 0 | 07-2012 | 108079 | 0 | 11-2017 | 97694 | 0 |
| 04-2007 | 114959 | 0 | 08-2012 | 107139 | 0 | 12-2017 | 97560 | 0 |
| 05-2007 | 108239 | 0 | 09-2012 | 94868 | 0 | 01-2018 | 109021 | 0 |
| 06-2007 | 110126 | 0 | 10-2012 | 95678 | 0 | 02-2018 | 86886 | 0 |
| 07-2007 | 119639 | 0 | 11-2012 | 100209 | 0 | 03-2018 | 120659 | 0 |
| 08-2007 | 124674 | 0 | 12-2012 | 91441 | 0 | 04-2018 | 106398 | 0 |
| 09-2007 | 106206 | 0 | 01-2013 | 102367 | 0 | 05-2018 | 108831 | 0 |
| 10-2007 | 106364 | 0 | 02-2013 | 78884 | 0 | 06-2018 | 98491 | 0 |
| 11-2007 | 114606 | 0 | 03-2013 | 107535 | 0 | 07-2018 | 103809 | 0 |
| 12-2007 | 109665 | 0 | 04-2013 | 97225 | 0 | 08-2018 | 105068 | 0 |
| 01-2008 | 108255 | 0 | 05-2013 | 96978 | 0 | 09-2018 | 95312 | 0 |
| 02-2008 | 90471 | 0 | 06-2013 | 86335 | 0 | 10-2018 | 94217 | 0 |
| 03-2008 | 123200 | 0 | 07-2013 | 97401 | 0 | 11-2018 | 100849 | 0 |
| 04-2008 | 118789 | 0 | 08-2013 | 97430 | 0 | 12-2018 | 96336 | 0 |
| 05-2008 | 113200 | 0 | 09-2013 | 87915 | 0 | 01-2019 | 107754 | 0 |
| 06-2008 | 107306 | 0 | 10-2013 | 86161 | 0 | 02-2019 | 90985 | 0 |
| 07-2008 | 123352 | 0 | 11-2013 | 88479 | 0 | 03-2019 | 113941 | 0 |
| 08-2008 | 115787 | 0 | 12-2013 | 87609 | 0 | 04-2019 | 110266 | 0 |
| 09-2008 | 106026 | 0 | 01-2014 | 90210 | 0 | 05-2019 | 106431 | 0 |
| 10-2008 | 108840 | 0 | 02-2014 | 83068 | 0 | 06-2019 | 97362 | 0 |
| 11-2008 | 105265 | 0 | 03-2014 | 99292 | 0 | 07-2019 | 112454 | 0 |
| 12-2008 | 110163 | 0 | 04-2014 | 94768 | 0 | 08-2019 | 106985 | 0 |
| 01-2009 | 90569 | 0 | 05-2014 | 91936 | 0 | 09-2019 | 97815 | 0 |
| 02-2009 | 120974 | 0 | 06-2014 | 88201 | 0 | 10-2019 | 98774 | 0 |
| 03-2009 | 125427 | 0 | 07-2014 | 95648 | 0 | 11-2019 | 102174 | 0 |
| 04-2009 | 120701 | 0 | 08-2014 | 94075 | 0 | 12-2019 | 102151 | 0 |
| 05-2009 | 110617 | 0 | 09-2014 | 87827 | 0 | 01-2020 | 91026 | 11791 |
| 06-2009 | 116439 | 0 | 10-2014 | 85996 | 0 | 02-2020 | 51506 | 68033 |
| 07-2009 | 123255 | 0 | 11-2014 | 85125 | 0 | 03-2020 | 73427 | 1730 |
| 08-2009 | 119965 | 0 | 12-2014 | 88397 | 0 | 04-2020 | 101262 | 995 |
| 09-2009 | 107161 | 0 | 01-2015 | 96649 | 0 | 05-2020 | 97651 | 143 |
| 10-2009 | 100504 | 0 | 02-2015 | 72869 | 0 | 06-2020 | 99319 | 517 |
| 11-2009 | 93627 | 0 | 03-2015 | 104427 | 0 | 07-2020 | 106135 | 803 |
| 12-2009 | 101113 | 0 | 04-2015 | 94350 | 0 | 08-2020 | 102304 | 721 |
| 01-2010 | 102470 | 0 | 05-2015 | 91194 | 0 | 09-2020 | 105377 | 356 |
| 02-2010 | 80143 | 0 | 06-2015 | 89224 | 0 | 10-2020 | 95633 | 583 |
| 03-2010 | 117048 | 0 | 07-2015 | 93586 | 0 | 11-2020 | 100561 | 545 |
| 04-2010 | 104691 | 0 | 08-2015 | 89228 | 0 | 12-2020 | 100209 | 529 |
