# Supplementary material for: Evaluating the impact of COVID-19 outbreak on hepatitis B and forecasting the epidemiological trend in mainland China: a causal analysis
Source: BMC Public Health. 2024 Jan 2;24:47. doi: 10.1186/s12889-023-17587-3 (PMC10763123; doi:10.1186/s12889-023-17587-3)
Supplement: Supplementary file 3 — Supplementary Material 3 [file 12889_2023_17587_MOESM3_ESM.docx]

**Table S3** Monthly average causal impact of the COVID-19 on hepatitis B notifications in 2020

| months | Average Cases | Predictions  (95%Cl) | Absolute  Effect  (95%Cl) | Cumulative Cases  (95%Cl) | Predictions  (95%Cl) | Absolute Effect  (95%Cl) | Relative Effect  (95%Cl) | *p* | Prob  Of Causal Effect |
| --- | --- | --- | --- | --- | --- | --- | --- | --- | --- |
| 1 | 91026 | 1e+05  [89901, 117512] | -10790  [-26486, 1125] | 91026 | 1e+05  [89901, 117512] | -10790  [-26486, 1125] | -11%  [-26%, 1.1%] | 0.056 | 94.000% |
| 1-2 | 71266 | 98258  [88086, 108578] | -26992  [-37312, -16820] | 142532 | 196515  [176172, 217156] | -53983  [-74624, -33640] | -27%  [-38%, -17%] | 0.004 | 99.573% |
| 1-3 | 76894 | 104806  [94853, 114519] | -27912  [-37625, -17959] | 230682 | 314417  [284560, 343557] | -83735  [-112875, -53878] | -27%  [-36%, -17%] | 0.009 | 98.130% |
| 1-4 | 82986 | 105907  [97295, 114670] | -22921  [-31684, -14309] | 331944 | 423626  [389179, 458679] | -91682  [-126735, -57235] | -22%  [-30%, -14%] | 0.005 | 99.548% |
| 1-5 | 85919 | 106416  [97983, 115758] | -20542  [-29839, -12064] | 429595 | 532303  [489915, 578791] | -102708  [-149196, -60320] | -19%  [-28%, -11%] | 0.004 | 99.574% |
| 1-6 | 88152 | 106464  [95986, 115589] | -18311  [-27437, -7834] | 528914 | 638782  [575919, 693534] | -109868  [-164620, -47005] | -17%  [-26%, -7.4%] | 0.005 | 99.487% |
| 1-7 | 90721 | 106895  [97238, 116069] | -16173  [-25348, -6517] | 635049 | 748262  [680669, 812485] | -113213  [-177436, -45620] | -15%  [-24%, -6.1%] | 0.004 | 99.590% |
| 1-8 | 92169 | 107997  [98901, 119104] | -15828  [-26935, -6732] | 737353 | 863975  [791208, 952833] | -126622  [-215480, -53855] | -15%  [-25%, -6.2%] | 0.004 | 99.578% |
| 1-9 | 93637 | 107705  [98196, 120608] | -14068  [-26971, -4559] | 842730 | 969343  [883764, 1085468] | -126613  [-242738, -41034] | -13%  [-25%, -4.2%] | 0.011 | 98.889% |
| 1-10 | 93836 | 105749  [95787, 117045] | -11912  [-23209, -1950] | 938363 | 1057485  [957867, 1170449] | -119122  [-232086, -19504] | -11%  [-22%, -1.8%] | 0.148 | 98.529% |
| 1-11 | 94448 | 106150  [94947, 117701] | -11702  [-23253, -499] | 1038924 | 1167649  [1044413, 1294707] | -128725  [-255783, -5489] | -11%  [-22%, -0.47%] | 0.026 | 97.414% |
| 1-12 | 94928 | 105692  [94691, 118057] | -10765  [-23129, 236] | 1139133 | 1268309  [1136297, 1416686] | -129176  [-277553, 2836] | -10%  [-22%, 0.22%] | 0.034 | 96.591% |
