# Supplementary material for: Evaluating the impact of COVID-19 outbreak on hepatitis B and forecasting the epidemiological trend in mainland China: a causal analysis
Source: BMC Public Health. 2024 Jan 2;24:47. doi: 10.1186/s12889-023-17587-3 (PMC10763123; doi:10.1186/s12889-023-17587-3)
Supplement: Supplementary file 4 — Supplementary Material 4 [file 12889_2023_17587_MOESM4_ESM.docx]

**Table S4** Time series of population, 2005-2020 China.

| year | Population (10000) | year | Population (10000) |
| --- | --- | --- | --- |
| 2005 | 1307560000 | 2013 | 1367260000 |
| 2006 | 1314480000 | 2014 | 1376460000 |
| 2007 | 1321290000 | 2015 | 1383260000 |
| 2008 | 1328020000 | 2016 | 1392320000 |
| 2009 | 1334500000 | 2017 | 1400110000 |
| 2010 | 1340910000 | 2018 | 1405410000 |
| 2011 | 1349160000 | 2019 | 1410080000 |
| 2012 | 1359220000 | 2020 | 1412120000 |
