# Supplementary material for: Evaluating the impact of COVID-19 outbreak on hepatitis B and forecasting the epidemiological trend in mainland China: a causal analysis
Source: BMC Public Health. 2024 Jan 2;24:47. doi: 10.1186/s12889-023-17587-3 (PMC10763123; doi:10.1186/s12889-023-17587-3)
Supplement: Supplementary file 5 — Supplementary Material 5 [file 12889_2023_17587_MOESM5_ESM.pdf]

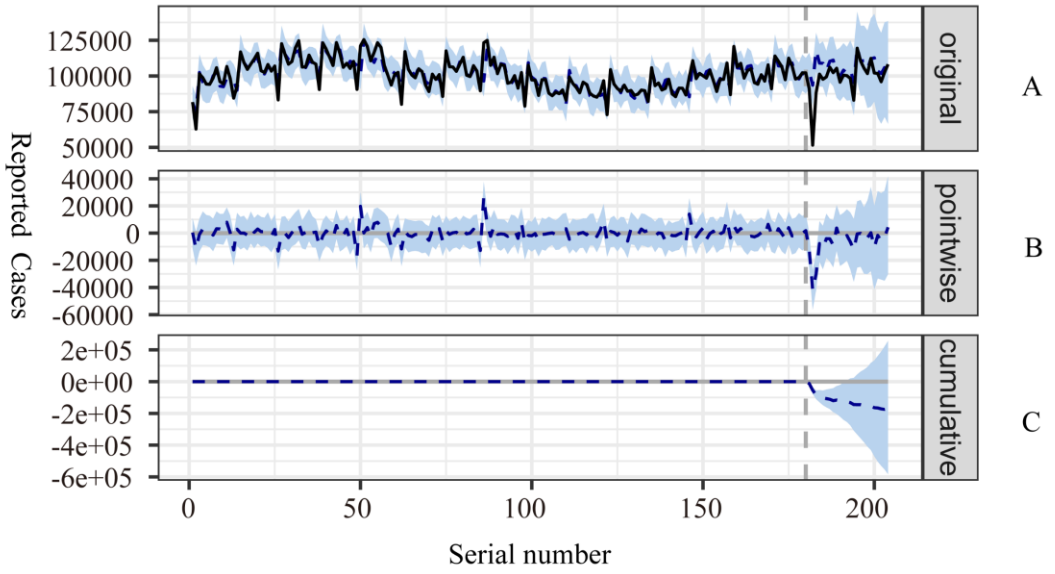

Fig. S1 Time series plot of causal effects of the COVID-19 on hepatitis B notifications in 2020-2021. The panel A, B and C shows the hepatitis B case notifications and counterfactual forecasted results for the post-outbreak period, pointwise causal effect and cumulative effect respectively.
